# Estimating Respirable Dust Exposure from Inhalable Dust Exposure

**DOI:** 10.1093/annweh/wxaa016

**Published:** 2020-02-28

**Authors:** Cornelia Wippich, Jörg Rissler, Dorothea Koppisch, Dietmar Breuer

**Affiliations:** Institute for Occupational Safety and Health of the German Soical Accident Insurance, Alte Heerstraße, Sankt Augustin, Germany

**Keywords:** aerosols, conversion functions, inhalable dust, occupational dust exposure, regression analysis, respirable dust, retrospective exposure assessment, workplace assessment

## Abstract

In the sector of occupational safety and health only a limited amount of studies are concerned with the conversion of inhalable to respirable dust. This conversion is of high importance for retrospective evaluations of exposure levels or of occupational diseases. For this reason a possibility to convert inhalable into respirable dust is discussed in this study. To determine conversion functions from inhalable to respirable dust fractions, 15 120 parallel measurements in the exposure database MEGA (maintained at the Institute for Occupational Safety and Health of the German Social Accident Insurance) are investigated by regression analysis. For this purpose, the whole data set is split into the influencing factors working activity and material. Inhalable dust is the most important predictor variable and shows an adjusted coefficient of determination of 0.585 (*R*^2^ adjusted to sample size). Further improvement of the model is gained, when the data set is split into six working activities and three material groups (e.g. *high temperature processing*, adj. *R*^2^ = 0.668). The combination of these two variables leads to a group of data concerned with *high temperature processing* with *metal*, which gives rise to a better description than the whole data set (adj. *R*^2^ = 0.706). Although it is not possible to refine these groups further systematically, seven improved groups are formed by trial and error, with adj. *R*^2^ between 0.733 and 0.835: *soldering*, *casting (metalworking)*, *welding*, *high temperature cutting*, *blasting*, *chiseling/embossing*, and *wire drawing*. The conversion functions for the seven groups are appropriate candidates for data reconstruction and retrospective exposure assessment. However, this is restricted to a careful analysis of the working conditions. All conversion functions are power functions with exponents between 0.454 and 0.946. Thus, the present data do not support the assumption that respirable and inhalable dust are linearly correlated in general.

## Introduction

Dust is a prevalent exposure at workplaces in various types of industries such as mining, foundries, chemical and food industries, stone working, and woodwork. Dust can consist of different materials like minerals, metallic and organic particles, which can differ greatly in size, shape, and density. Depending on the aerodynamic diameter, the particles can reach various regions of the respiratory tract and are assigned to the inhalable, thoracic, or respirable dust fraction (European Committee for Standardization (1993), EN 481:1993-09; ISO 7708:^1995^; WHO, 1999). The largest particles can be inhaled and are deposited in the air passages of the extrathoracic region between the mouth, the nose, and the larynx (WHO, 1999). International standards (EN 481:1993-09; ISO 7708:^1995^) define the mass fraction of inhalable particles by the separation function I=50*(1+exp[-0,06*D]), where *I* is the percentage of particles with an aerodynamic diameter of *D* in µm. This convention is defined for *D* ≤ 100 µm. In other words, the inhalable dust fraction consists of particles with an aerodynamic diameter up to 100 µm (ISO 7708:^1995^; European Committee for Standardization (2014a,b), EN 13205-2:2014a,b). Smaller particles are able to reach the gas-exchange region of the lungs and form the respirable dust fraction. In words of particle size, the limit for entering the alveolar region is between 10 and 15 µm (WHO, 1999; EN 13205-2:2014a,b).

If dust particles cannot be exhaled or cleared from the respiratory tract, they can remain at the same location for a long time and may cause serious harm. Adverse health effects caused by dust comprise, for example, allergic reactions, pneumoconiosis (especially silicosis), cancer, and heart diseases (Verma, 1984; WHO, 1999; Baur, 2013). Often the inhaled particles imply additional risks because of hazardous substances. Metal dusts frequently contain toxic compounds like lead, mercury, nickel, chromium, or cadmium, which can cause pulmonary fibrosis and dyspnea for example (WHO, 1999; Bender, 2005).

With the knowledge of these health-related effects caused by occupational dusts of different size, measuring different dust fractions in work environments has gained further importance for the evaluation of exposure and risk to workers over the last few years. Historically, dust measurements in Germany have mainly targeted the inhalable dust fraction, which has been measured and evaluated according to international standards (EN 481:1993-09; ISO 7708:^1995^). The introduction of the legal limit value (maximum workplace concentration [MAK]) for respirable dust in the year 1973 and subsequent lowering of occupational exposure limits in Germany (Barig and Blome, 1999; Hahn and Möhlmann, 2011; Ausschuss für Gefahrstoffe, 2014) have spurred measurements of the respirable fraction, with concomitant increase in the amount of available exposure data. So in the early years of dust measurement mainly inhalable dust was sampled, whereas the amount of respirable dust measurements increased after the introduction of the limit value, exceeding the yearly number of measurements of inhalable dust resulting in a higher number of data for respirable dust. The increase in measurements of respirable dust was not unique in Germany, there was also an international trend in measuring more than the inhalable dust fraction. This was also caused by the advances of sampler technology. While the assessment of current exposures has improved, the retrospective assessment of the exposure to respirable dust remains problematic, if only historical data for inhalable dust are available. Therefore, a possibility to convert the measured concentration of inhalable dust into respirable dust concentration mathematically is highly desirable for the hazard assessment or in the investigation of occupational diseases. Further problems occur for epidemiological studies especially when these studies are used to derive limit values.

Various studies have contributed to discussions which are concerned with the occurrence of different dust fractions in selected types of industries. These studies often compare conversion factors between ‘total’ and ‘inhalable’ dust in specific types of industries (Tsai *et al.*, 1995; Vinzents et al., 1995; Werner *et al.*, 1996; Tsai *et al.*, 2011), or the performances of different measurement systems are compared (Lilienberg and Brisman, 1994; Linnainmaa *et al.*, 2007; Martin and Zalk, 2011). Only a limited number of studies have focused on the conversion of inhalable to respirable dust. A study by Dahmann *et al.* (2007) attempted to reconstruct the exposure of inhalable and respirable dust, crystalline silica and heavy metals in former uranium mines by performing parallel measurements with original sampling equipment, instead of calculating the dust concentrations with the aid of a conversion function. Notø *et al.* (2016) determined a ratio of 0.085 for respirable to inhalable dust in cement production industry. Another study (Hauptverband der gewerblichen Berufsgenossenschaften, 1996) identified ratios of respirable to inhalable dust for specific working activities such as grinding gypsum (0.19), grinding and transporting quartz sand (0.26), clay processing (0.20), and loading cement (0.21). Also, the exposure to inhalable and respirable particles in welding fume (Lehnert *et al.*, 2012) and specific workplaces of different crematoria (Korczynski, 2011) have been investigated. From these few examples, it can be seen that *working activity* and *material* are important variables in defining a relation between inhalable and respirable dust. Most of these studies assume a linear relationship and calculate conversion factors.

This study analyzes the nonpublic database MEGA of exposure data obtained by the surveillance activity of the German Social Accident Insurance (Gabriel *et al.*, 2010). MEGA was established in 1972 and is designed for the evaluation of occupational diseases, hazard and exposure analysis in specific working areas, as well as time-dependent analysis of exposure to hazardous substances at working places. The database holds over 3 million data sets with exposures to about 870 hazardous substances including information of measurement systems used, working conditions, analytical methods, and characteristics of measurement sites. Publications of statistical evaluations of the MEGA database can be found under https://www.dguv.de/ifa/gestis/expositionsdatenbank-mega/expositionsdaten-aus-mega-in-publikationen/index-2.jsp.

The dust exposure data in the MEGA database are analyzed in this study in order to determine a possible relation between inhalable and respirable dust measurement results depending on working environments and materials.

## Materials and methods

### Data selection

The MEGA database contains independent data sets for measurements of inhalable and respirable dust. This study starts with records from 1961 to 2016 which contain 103 825 data sets for inhalable dust and 222 501 data sets for respirable dust.

First, measurements are excluded, if

- the measurement duration is < 2 h,
- a concentration is below the limit of quantification, and
- the measured concentrations for inhalable dust are >100 mg m^−3^ or for respirable dust are >10 mg m^−3^.

With these restrictions a total of 26 337 pairs of inhalable and respirable dust were excluded. The limits for the measurement duration and the range of concentration lead to values that are representative for the working conditions. The effect of including samples above the concentration cutoff values is discussed in Results. According to the European standard EN 689:1995 the minimum number of samples which have to be taken during a work shift with constant exposure is dependent on the sampling duration. When the sampling duration is higher or equal 2 h, one measurement is sufficient (European Committee for Standardization (1995), EN 689:1995).

Secondly, pairs of inhalable and respirable measurements are formed if:

- the measurement has been performed at the same day and time (starting and ending times of both measurements do not differ by more than 5 min),
- the measurements have the same industrial sector, report number, type of sampling, and working activity,
- the respirable dust concentration *c*_R_ is not higher than the concentrations of inhalable dust *c*_I_.

With these criteria further 2704 pairs of inhalable and respirable dust were excluded. The industrial sector describes the type of industry where the measurements are performed, such as the mining industry, production of concrete products, foundries, or the ceramic industry. The variable *working activity* combines the task and the process. The type of sampling describes if the sample was taken by personal or stationary sampling. For the personal sampling, the exact position of the system is also described, for example, behind the welding protection shield or in front of the face protection (if applicable).

Although the pairs of inhalable and respirable dust are not previously linked in the database, the risk of forming wrong pairs is very low. The pairs are formed systematically with the help of 12 variables, for example:

- Same factory
- Same location within the factory
- Same day
- Same starting and ending time

The software-based systematic pairing was also verified by the first author for a random subsample. Because wrong pairing of measurements would lead to wrong ratios of the dust fractions and in the worst case to incorrect conversion functions, special attention was paid to this crucial point of the study.

Respirable dust is a subset of the inhalable dust. Therefore, measurements with *c*_R_ > *c*_I_ can be caused by incorrect sampling, spatial variability of the dust concentrations, or could result from particle movement and thermal effects. This criterion only affects 592 pairs of measurements.

If one merges the data sets of respirable and inhalable dust fractions by considering the described requirements, it is possible to form a new data set consisting of 15 120 pairs gathered between the years 1989 and 2016. The data used are collected in 818 different industrial sectors. The majority of dust concentration values is recorded in 2-h measurements (*n* = 9648).

### Measurement systems

Table 1 lists the most commonly used sampling systems for the parallel measurements of inhalable and respirable dust. As additional information the sampling rate of each system and sampling type is given.

**Table 1.** Sampling systems and sampling rates used for both dust fractions in parallel measurements.

Sampler inhalable dust (sampling rate)	Sampler respirable dust (sampling rate)	*n*	Type of sampling
VC-25 G (375 l min−1)	VC-25 F (375 l min−1)	3788	Stationary
VC-25 G (375 l min−1)	PM4-F (66.7 l min−1)	169	Stationary
GSP (3.5 l min−1)	FSP-10 (10 l min−1)	1298	Stationary
GSP (3.5 l min−1)	FSP-10 (10 l min−1)	5273	Personal
GSP (3.5 l min−1)	FSP-2 (2 l min−1)	495	Personal
GSP-10 (10 l min−1)	FSP-10 (10 l min−1)	854	Stationary
GSP-10 (10 l min−1)	FSP-10 (10 l min−1)	1822	Personal
GSP-10 (10 l min−1)	PM4-F (66.7 l min−1)	155	Stationary
PM4-G (66.7 l min−1)	PM4-F (66.7 l min−1)	799	Stationary

All samplers used in this study are validated according to the international standards EN 13205 and European Committee for Standardization (2012), EN 1540 for sampler performance testing systematic deviation of the sampler, measurement uncertainty, measuring range, precision, and impact of the main influential variables (e.g. particle size, composition of particles, aerosol mass, and variations in the sampling rate) (European Committee for Standardizsation (2014a,b), EN 13205-1:2014a,b). In addition, the use of validated measurement systems is a compulsory requirement of the MEGA database.

The samplers VC-25 and PM4 can only be used for stationary measurements. The samplers GSP and FSP can be used for both stationary and personal measurements (Mattenklott and Möhlmann, 2011). The VC-25 and PM4 samplers are used with two different sampling heads. In Table 1 these sampling heads are characterized with ‘G’ for inhalable dust and ‘F’ for respirable dust. The VC-25 G and PM4-G collect dust through a ring slit orifice with an aspiration speed of 1.25 m s^−1^ independent from the sampling rate and the orientation (Coenen, 1981; Riediger, 2001). For inhalable dust particles with an aerodynamic diameter of 10 µm are collected with the VC-25 G to about 80%, with 20 µm to about 70% and with 50 µm to about 55% (Coenen, 1981). Particles which are sampled with the VC-25 F are collected through a ring slit and the separation of respirable dust fraction is performed via impaction of large particles (Siekmann, 1998). The separation of the respirable dust fraction using the PM4-F sampler is done using a cyclone preseparator (Siekmann, 1998). With the comparably high sampling rates of VC-25 and PM4, lower limits of detection can be achieved (Möhlmann, 2005).

The VC-25 is also used as reference method for inhalable dust measurements (Riediger, 2001). The GSP-sampling heads for sampling rates at 3.5 and 10 l min^−1^, respectively, were constructed to achieve the maximum compliance with the reference method (VC-25 G) (Riediger, 2001).

It is in principle possible that, within the limits set by the validation standards, some measurement systems are more sensitive than others. However, if all systems are applied with the same probability in all measurement situations, these differences will not affect the average values of the analysis. Therefore, it has been confirmed by visual inspection of scatterplots, that the application of the measurement systems is evenly distributed across all working activities and all measurement departments. Since the latter are focused on certain dust materials, this is an indicator that also the material groups are not biased by the use of measurement equipment.

### Statistical and mathematical methods

All statistical analyses are performed using the statistical software IBM SPSS statistics, version 23 (IBM Corp.). All tests which are mentioned in this section are described in statistics texts (Sachs, 1999; Janssen and Laatz, 2017). For all tests, the significance level is fixed at *α* = 0.05.

For the concentration measurements of this study, the hypothesis of a log-normal distribution cannot be rejected at the significance level of 0.05 using the Lilliefors-corrected Kolmogorov–Smirnov test (Sachs, 1999). This is in accordance to other studies (Burstyn *et al.* 1997; Andersson *et al.*, 2009; Lehnert *et al.*, 2012; Weggeberg *et al.*, 2016), and, therefore, this study assumes a correlation between ln(*c*_R_) (natural logarithm of the respirable dust concentration) and ln(*c*_I_) (natural logarithm of the inhalable dust concentration):

$$\begin{array}{l} ln(c_{R})=k\cdot ln(c_{I})+C_{0}, \end{array}$$ (1)

where *k* and *C*_0_ are the slope and the intercept, which can be determined by a regression analysis. The results for *k* and *C*_0_ are given with their standard errors (compare results, Table 2). More important for retrospective analyses is the standard error of the fitted regression function *s*_Fit_(ln(*c*_R_)). This can be used to calculate confidence intervals for the regression function at a given ln(*c*_I_) (Draper and Smith, 1998). The smallest *s*_Fit_ values are obtained for the mean value of ln(*c*_I_) and the largest values are obtained at the extreme values of ln(*c*_I_). Therefore, we give the range of *s*_Fit_ for every regression analysis.

**Table 2.** Regression coefficients *k*, *C*_0_ with standard errors for equation (1), range of standard errors for regression function *s*_Fit_(ln(*c*_R_)) within groups 1–6 for *working activity*, groups A–C for *material*, combined groups of *working activities and material*, and heuristic groups α–η including group names as defined in Table 3.

ID	Group	*n*	*R*	*adj. R* ^2^	*C*_0_	*k*	*s*_Fit_(ln(*c*_R_))	Conversion function
0	Entire data set	15 120	0.765	0.585	−0.990 ± 0.006	0.594 ± 0.004	0.0092–0.0400	$c(R)=c{\left(I\right)}^{0.594}*e^{-0.990}$
*Working activities*								
1	Surface treatment	805	0.735	0.540	−1.046 ± 0.024	0.500 ± 0.016	0.0427–0.1396	$c(R)=c{\left(I\right)}^{0.500}*e^{-1.046}$
2	High temperature processing	2974	0.818	0.668	−0.751 ± 0.011	0.729 ± 0.009	0.0184–0.0708	$c(R)=c{\left(I\right)}^{0.729}*e^{-0.751}$
3	Filling/transport/storage	3473	0.791	0.626	−1.093 ± 0.012	0.586 ± 0.008	0.0192–0.0698	$c(R)=c{\left(I\right)}^{0.586}*e^{-1.093}$
4	Machining/abrasive techniques	4640	0.776	0.602	−1.031 ± 0.016	0.578 ± 0.013	0.0169–0.0553	$c(R)=c{\left(I\right)}^{0.578}*e^{-1.031}$
5	Forming	1348	0.774	0.599	−1.037 ± 0.011	0.579 ± 0.007	0.0272–0.1071	$c(R)=c{\left(I\right)}^{0.579}*e^{-1.037}$
6	Others	1880	0.768	0.590	−1.100 ± 0.016	0.593 ± 0.011	0.0275–0.1106	$c(R)=c{\left(I\right)}^{0.593}*e^{-1.100}$
*Material*								
A	Mineral-dominated	9315	0.785	0.616	−1.058 ± 0.007	0.581 ± 0.005	0.0119–0.0512	$c(R)=c{\left(I\right)}^{0.581}*e^{-1.058}$
B	Metal-dominated	5269	0.748	0.559	−0.851 ± 0.010	0.614 ± 0.008	0.0146–0.0531	$c(R)=c{\left(I\right)}^{0.614}*e^{-0.851}$
C	Fiber-dominated	536	0.761	0.578	−1.176 ± 0.031	0.614 ± 0.023	0.0543–0.1977	$c(R)=c{\left(I\right)}^{0.614}*e^{-1.176}$
*Combined groups*								
(1-A)	Surface treatment—mineral-dominated	540	0.756	0.571	−1.043± 0.059	0.512 ± 0.038	0.0549–0.1660	$c(R)=c{\left(I\right)}^{0.512}*e^{-1.043}$
(2-B)	High temperature processing—metal-dominated	2265	0.840	0.706	−0.687 ± 0.013	0.758 ± 0.010	0.0268–0.0961	$c(R)=c{\left(I\right)}^{0.758}*e^{-0.687}$
(4-A)	Machining/abrasive techniques—mineral-dominated	2632	0.802	0.643	−1.015 ± 0.026	0.595± 0.017	0.0227–0.0732	$c(R)=c{\left(I\right)}^{0.595}*e^{-1.015}$
(6-B)	Other—metal-dominated	331	0.779	0.608	−0.898 ± 0.068	0.618 ± 0.054	0.0608–0.1910	$c(R)=c{\left(I\right)}^{0.618}*e^{-0.898}$
*Heuristic groups*								
α	Soldering	34	0.917	0.835	−0.559 ± 0.074	0.946 ± 0.073	0.1634–0.4417	$c(R)=c{\left(I\right)}^{0.946}*e^{-0.559}$
β	Casting (metalworking)	77	0.877	0.767	−0.430 ± 0.058	0.913 ± 0.049	0.0857–0.2322	$c(R)=c{\left(I\right)}^{0.913}*e^{-0.430}$
γ	Welding	1126	0.875	0.766	−0.601 ± 0.018	0.803 ± 0.014	0.0297–0.1005	$c(R)=c{\left(I\right)}^{0.803}*e^{-0.601}$
δ	High temperature cutting	176	0.897	0.803	−0.716 ± 0.028	0.750 ± 0.028	0.0832–0.2832	$c(R)=c{\left(I\right)}^{0.750}*e^{-0.716}$
ε	Blasting	57	0.907	0.819	−1.107 ± 0.080	0.724 ± 0.045	0.1847–0.4479	$c(R)=c{\left(I\right)}^{0.724}*e^{-1.107}$
ζ	Chiseling, embossing	41	0.912	0.827	−1.264 ± 0.111	0.695 ± 0.050	0.2113–0.4946	$c(R)=c{\left(I\right)}^{0.695}*e^{-1.264}$
η	Wire draw	61	0.859	0.733	−1.028 ± 0.087	0.695 ± 0.054	0.1387–0.4000	$c(R)=c{\left(I\right)}^{0.695}*e^{-1.028}$

One can transform equation (1) back into a function of the original concentrations:

$$\begin{array}{l} c_{R}=c_{I}^{k}\cdot e^{C_{0}}. \end{array}$$ (2)

Moreover, one can see in equation (2) that *c*_R_ tends to zero, if *c*_I_ tends to zero. This is a necessary condition, since *c*_R_ ≤ *c*_I_. Also note that the assumption of a linear relation between *c*_R_ and *c*_I_ is included in equations (1) and (2), if the value 1 is included in the 95% confidence interval of *k*.The worst-case assumption *c*_R_ = *c*_I_ is included, if *C*_0_ = 0 and *k* = 1.

In principle, it is possible to expand equation (1) with further (linear) terms for other independent variables, for example the *working activity* and the *material*. However, it is self-evident that *c*_I_ is influenced by the *working activity* and the *material*. Therefore, a multilinear regression analysis is not possible, which assumes the independence of its variables. The *measurement system* has been ruled out as variable in the preceding section and it has been confirmed also that the *year of the measurement* has no influence on the measured concentrations (see Results).

**Figure 1.**
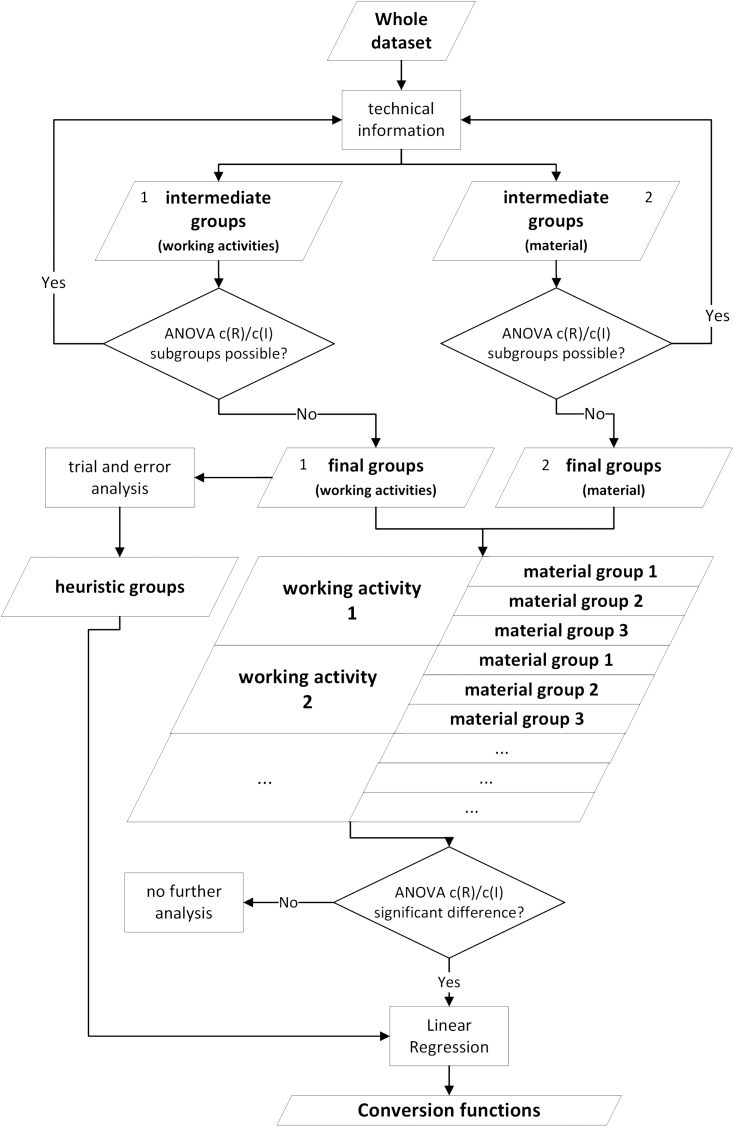
Flowchart of the group formation steps and statistical tests (for each group distribution: Kolmogoroff–Smirnov, ANOVA: *F*-test, Kruskal–Wallis test, variance homogeneity: Levene-test and graphic evaluation, *post hoc* tests: Games-Howell).

It is necessary to form mutually independent groups of measured dust concentrations for *working activity* and *material*. Within these groups a regression analysis (equation (1)) is possible. The criterion to form these groups is primarily based on the technical information available in the database. The group formation steps, as well as the statistical tests are shown in the flowchart (Fig. 1). The data are divided into groups with different *working activities* on the basis of technical specifications for production processes (Deutsches Insitut für Normung (2003) (DIN) DIN 8580:^2003^) or the attributed energy content of the process (e.g. welding or the use of fast rotating abrasive tools). In the next step the whole data set is divided into groups with different *material*. In a following step, working activity and material groups are combined (Fig. 1).

This systematic procedure leads to groups of paired measurement that are subjected to a linear regression analysis (equation (1)). The residuals of all analyses have been checked graphically for normality (histograms) and the absence of trends: There were no patterns discernible in the residuals apart from the omission of *c*_R_ > *c*_I_, and all residuals were normally distributed. In addition, the absence of autocorrelation has been confirmed by performing the Durbin–Watson test (Sachs, 1999). The quality of the regression parameters is measured by the correlation coefficient *R* and the adjusted coefficient of determination *R*^2^ (Janssen and Laatz, 2017):

$$\begin{array}{l} adj.\;R^{2}=R^{2}\frac{m}{n-m-1}(1-R^{2}). \end{array}$$ (3)

This accounts for the number of variables *m* and the number of paired data *n*. Since in our case *n* >> *m*, this leads to adj. *R*^2^ ≈ *R*^2^.

Apart from the groups that have been identified in this systematic way, it is also possible to find groups of data pairs which show a better correlation (higher adj. *R*^2^) than the data of the systematic groups. They have a more restrictive definition of *working activity* or *material*. Since these groups are identified by trial and error, they are denoted heuristic groups (compare Fig. 1). For the construction of these groups, single *working activities* were combined within groups 1–6 (compare Table 2) if concerning the same type of activity (e.g. different welding processes). They were than pooled into one heuristic group if regression coefficients were similar and if adj. *R*^2^ was larger than adj. *R*^2^ for the groups 1–6.

## Results

### Year of measurement

**Figure 2.**
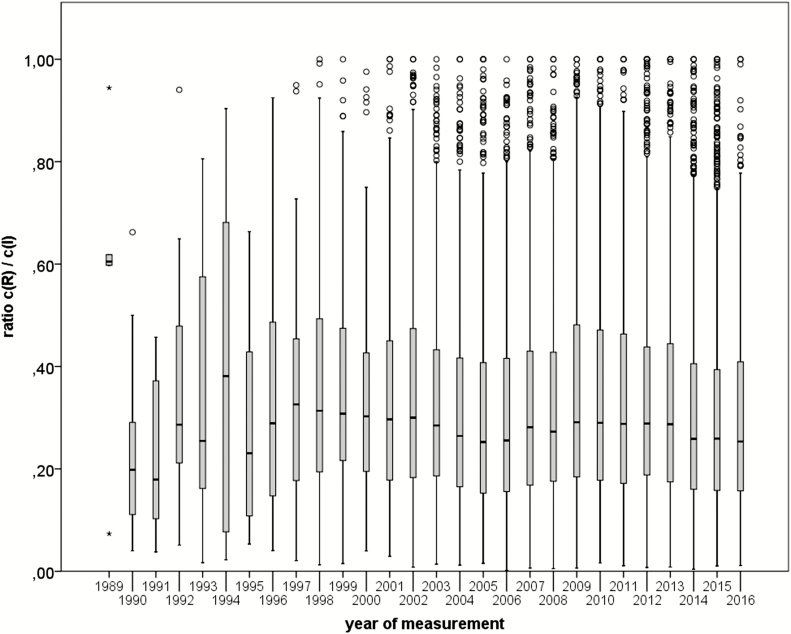
Boxplot of ratios *c*(R)/*c*(I) for the years 1989–2016.

Fig. 2 shows boxplots of the ratios *c*_R_/*c*_I_ for the years 1989–2016. Only 227 parallel measurements are available from the years 1989–1997; each of these years contains not more than 57 measurements, and often <20. This leads to the large variations observed in this time period. The remaining 14 893 parallel measurements are recorded in the years 1998–2016, and the boxplots of the ratio *c*_R_/*c*_I_ show mainly no variation.

Due to the small number of data, the results for years 1989–1997 are considered negligible, and the exposure appears homogenous in the remaining time periods. The small variation cannot be explained by technical arguments such as introduction of new samplers or the decline of the mining industry in Germany. Analysis of Variance (ANOVA) analyses only lead to spurious differences in the median from the years 2004–2006 and 2014–2016. These median differences are small effects that manifest as significant results in ANOVA due to the large amount of data and were considered to occur by chance (fallacy of large sample size).

For these reasons, we postulate homogeneous exposure ratios *c*_R_/*c*_I_ over the time periods studied and exclude the *years of measurement* as independent variable from the analysis. However, one has to stress that the use of the conversion functions is, in principle, limited to inhalable dust concentrations, which are similar to those in Germany between the years 1998–2016.

### Inhalable dust

Using simple linear regression for the whole data set of 15 120 paired measurements, where just the results for inhalable dust are taken into account as a predictor variable, one obtains *k* = 0.594 and *C*_0_ = −0.990 in equation (1). The adjusted coefficient of determination and correlation coefficient show values of 0.585 and 0.765, respectively.

**Figure 3.**
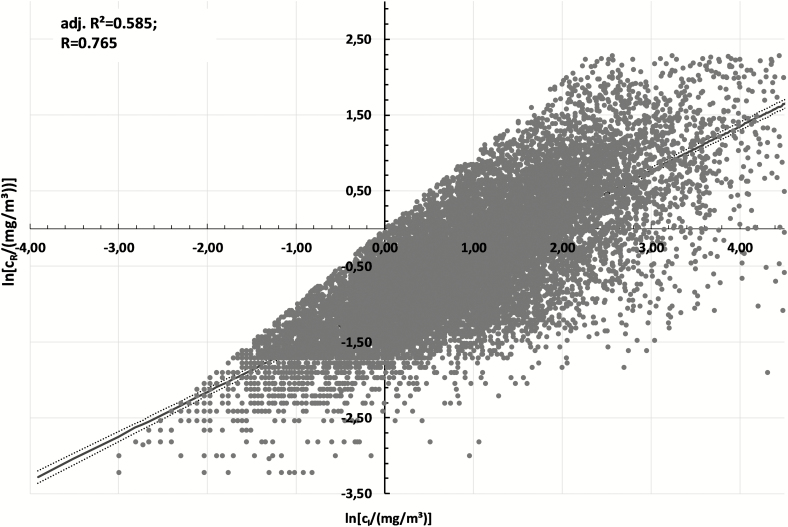
Scatterplot *y* = ln(*c*(R)) versus *x* = ln(*c*(I)) with linear regression line and the 95th confidence interval (equation (1)).

In Fig. 3 one can see a scatterplot of all parallel measurements with log-transformed values and the linear regression in the 95% confidence interval. The cutoff values due to the data selection for *c*_R_ > *c*_I_, *c*_R_ > 10 mg m^−3^ (ln(10) ≈ 2.3), *c*_I_ > 100 mg m^−3^ (ln(100) ≈ 4.6) are clearly visible.

There are only 119 sample pairs with concentrations above these cutoff that fulfill also the other selection criteria. As expected, the inclusion of such a small number of samples does not have a large impact on the analysis at this stage: the correlation coefficient *R* increases only by 0.005 (the adj. *R*^2^ only by 0.008). However, to include these samples would introduce a bias the analysis toward a nonrepresentative exposure condition. Therefore, these values remain excluded.

#### Working activity

The whole data set can be divided into six mutually independent groups according to the systematic procedure outlined in Materials and methods:

- Group 1: *surface treatment* (such as e.g. glazing, spray painting, powder coating, and galvanization)
- Group 2: *high temperature processing* (such as e.g. thermal cutting, extrusion, soldering, and welding)
- Group 3: filling/transport/storage
- Group 4: machining/abrasive techniques
- Group 5: *forming* (such as e.g. roll forming, pressing, and bending)
- Group 6: *others* (contains all other working activities).

The groups have been formed on the basis of technical data available in the database in connection with specifications (DIN 8580:^2003^). Each group combines different working activities which, unfortunately, cannot be resolved further in a systematic way.

In the next step, the data pairs within groups 1–6 are subjected to a linear regression analysis. The dominant result is that the coefficients for group 2, *k* = 0.729 and *C*_0_ = −0.751, differ strongly from the coefficients of the other groups; the differences are much larger than the respective standard errors (Table 2). To a lesser extent differences are also seen between group 1 in comparison to groups 3–6. The values of *s*_Fit_ mainly reflect the different *n*.

While for group 1 the correlation coefficient decreases with respect to the total data set, only a slight increase is observed for groups 3–6. Only group 2 yields a clearly better description of the data with *R* = 0.818 (Table 2).

#### Material

As in the preceding section, the whole data set is divided into mutually independent groups, now for the criterion *material*. This division is again based on technical information available in the database. At first, 12 material groups are formed which are unbalanced in numbers. They are subsequently pooled in three larger groups:

- Group A: mineral-dominated
  1. synthetic material/rubber/epoxy resin/powder coating (*n* = 799)
  2. mineral material/glass/plaster/gypsum/concrete/carbon/graphite (*n* = 7576)
  3. *others* (*n* = 940)
- Group B: metal-dominated
  1. metal/metal ores/slag/metallic shot (*n* = 5069)
  2. lacquers/paint (*n* = 108)
  3. electronic waste (*n* = 92)
- Group C: fiber-dominated
  1. textile (*n* = 101)
  2. mineral fibers/ceramic fibers (*n* = 197)
  3. *paper* (*n* = 126)
  4. asphalt/bitumen (*n* = 112)

Since *lacquers/paint* is mainly concerned with polishing and grinding of metallic surfaces and *electronic waste* is mainly concerned with metallic waste, it is reasonable to combine them in group B. Also, *asphalt/bitumen* belongs to the *fiber* group because it is mainly concerned with coating of fibrous materials using asphalt or bitumen.

The values for the regression coefficients are roughly similar to the values of the total data set, and the *metal*- and *fiber-dominat*ed groups have an identical *k* = 0.614. In addition, only the mineral-dominated group A shows a better description of the data in comparison with the total data set (*R* = 0.785, Table 2). The standard errors for *mineral-* and *metal-dominated* groups for *k*, *C*_0_ are of the same order of magnitude as for the *working activity* groups of the preceding section. The larger standard errors for the *fiber-dominated* group can be attributed to the smaller *n* and a concomitantly larger standard error. Also *s*_Fit_ shows the same dependence on *n* as for the groups 1–6.

#### Working activity and material

In a third step, the definitions for *working activity* and *material* are combined. To this end, the groups 1–6 are divided into three *material* groups using the definitions of the preceding section.

From the total of 18 groups only 9 groups showed an increased adj. *R*^2^. From these nine groups, the increase in adj. *R*^2^ was either smaller than 0.01 (three groups), or the group size was smaller than 50 with values from very different processes (two groups). Therefore, only four groups were selected for further discussion:

- surface treatment—mineral-dominated (1-A)
- high temperature processing—metal-dominated (2-B)
- machining/abrasive techniques—mineral-dominated (4-A)
- other—metal-dominated (6-B)

The increase in standard errors in comparison to groups 1–6 or A–B can be attributed to the reduced number of data pairs in each group (Table 2). The coefficients *k*, *C*_0_ of group 1A are very similar to those of group 1, and the adj. *R*^2^ is still smaller than for the total data set. For group 6-B, the increase in adj. *R*^2^ compared to group 6 is small and the group only contains 331 data pairs of very different processes.

The groups 2-B and 4-A are different, since they both have more than 2000 data pairs. Although they represent 57–76% of the respective *working activity* group, they have different *k* values than the underlying *working activity* groups. This indicates that the formation of subgroups really improved the description. In addition, they show the largest increase in the adj. *R*^2^ for the combined groups (>0.04). The best result of the systematic analysis is group 2-B, which shows a higher adj. *R*^2^ than the total data set (adj. *R*^2^ = 706). Unfortunately, it is not possible to improve these groups further in a systematic way.

#### Heuristic groups

Apart from the systematic approach described above, it was possible to identify some smaller subgroups by trial and error (Table 3), which improved the correlation.

**Table 3.** Heuristic groups with listed special activities, materials and number of data pairs (*n*).

ID	Group name	Originating group no.	Working activities	Material	*n*
α	Soldering	2-B	Soft soldering/soft soldering, flame soldering/hard soldering, flame soldering/arc soldering, MIG soldering	Metal	34
β	Casting (metalworking)	2-B	Hot/-cold-chamber die-casting machine or plant/continuous casting machine or plant	Metal	77
γ	Welding	2-B	Manual arc welding with and without coated rod electrode/metal inert gas welding/metal active gas welding/tungsten inert gas welding/arc welding, mixed arc process/plasma welding/laser welding/ resistance spot welding/metal welding, mixed welding processes	Metal	1126
δ	High temperature cutting	2-B	Flame cutting/plasma cutting/laser cutting	Metal	176
ε	Blasting	1	Treatment and post-treatment blasting/fettling shop, abrasive blasting,silica sand abrasive, workpiece sand-coated, room/abrasive blasting systems, dry, open/sandblasting	All materials	57
ζ	Chiseling, embossing	4-A	Chiseling, manually/chiseling, mechanically/embossing, manually/embossing, mechanically	Mineral	41
η	Wire drawing	2-B	Wire drawing	Metal	61

Most of the heuristic groups are subgroups of group 2-B and are concerned with special activities of high temperature processing with metals (groups α, β, γ, δ, and η). Only blasting (group ε) is a subgroup of group 1 and chiseling (group ζ) is a subgroup of group 5-A. Apart from welding (group γ) the number of data pairs in each group is much smaller than in the preceding sections.

The regression models in Table 2 for the heuristic groups give better descriptions of the data than those of the systematic approach. The adj. *R*^2^ range from 0.733 to 0.835 and *R* from 0.859 to 0.917. The standard errors of the coefficients increase according to the decreasing group size. The standard errors of the fit function *s*_Fit_ also increase with the decreasing group size, however, to a lesser extent than expected due to the better description of the data set.

**Figure 4.**
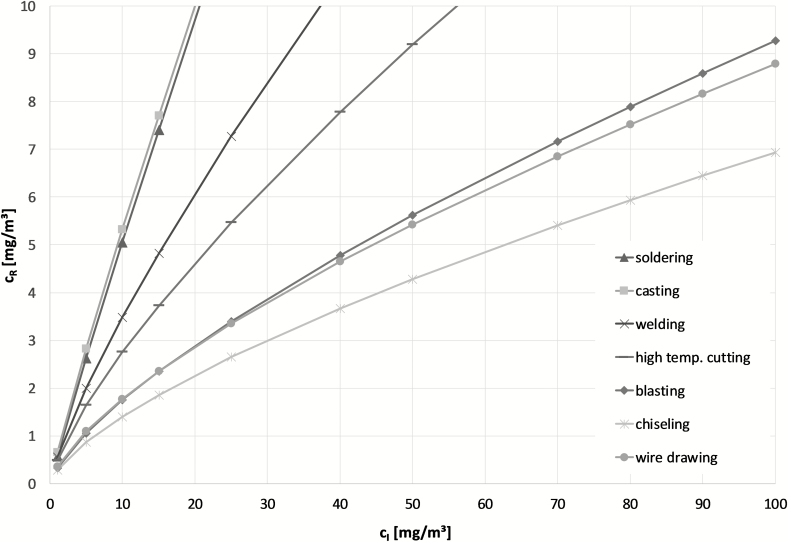
Comparison of the determined conversion functions for the heuristic groups without real measured concentrations.

Fig. 4 shows plots of equation (2) using the coefficients *k*, *C*_0_ for groups α–η. At first, one has to acknowledge the large variety of the groups that originate from group 2-B. The groups *casting* and *soldering* are almost indistinguishable from a linear relation (*k* ≈ 1 for groups α and β), while wire drawing shows a much smaller *k* (*k* = 0.695) with a similar correlation coefficient. In addition, there is now a large variety for both, in *k* (0.695 ≤ *k* ≤ 0.946) and in *C*_0_ (−1.264 ≤ *C*_0_ ≤ −0.430).The effect of a smaller intercept can be seen by comparing groups ζ (chiseling, embossing) and η (wire drawing), which have identical *k*. However, the graph of group ζ is less steep due to a smaller *C*_0_. It can be seen from Fig. 4 that each heuristic group shows a different conversion function and if one measures, for example *c*_I_ = 10 mg m^−3^, the result for *c*_R_ is different in each group, such as *c*_R_ ≈ 1.5 mg m^−3^ for ζ (chiseling and embossing) or *c*_R_ ≈ 5.0 mg m^−3^ for *α* (soldering).

## Discussion

### Application of equation (1) or (2)

Let us first examine two limiting cases of equation (1):

(1) The worst-case assumption *c*_R_ = *c*_I_, which is equivalent to *C*_0_ = 0 and *k* = 1.
(2) The linear assumption for *c*_R_ < *c*_I_, which is equivalent to *C*_0_ < 0 and *k* = 1.

The worst-case assumption has not been observed in our data set. In addition, all *C*_0_ values throughout this study are negative (−0.430 ≤ *C*_0_ ≤ −1.264), which is necessary to avoid unphysical values (*c*_R_ > *c*_I_) in the analyzed data range, if *k* ≠ 1.

Moreover, all *k* values in this study are smaller than 1 (0.454 ≤ *k* ≤ 0.946), although the regression analysis does not prohibit *k* > 1. This indicates that *k* < 1 is indeed a systematic effect. Which means that the resulting curve is not linear and that the ratio *c*_R_/*c*_I_ is declining with increasing values of *c*_I_. From Tables 2 and 3, for example, one can deduce that group 2-B is a superposition of data originating all from groups like α, β, γ, δ, and η, which all have *k* ≤ 1. Although this is not a rigorous proof, it is unlikely from this study to assume a purely linear relation between *c*_R_ and *c*_I_.

One could argue, that the value of one is included in the confidence interval of *k* for groups α and β, that is, one cannot exclude that the limiting case of *k* = 1 is actually valid for these two groups. However, a close inspection of Fig. 4 reveals a nonlinear pattern in the data. This nonlinear behavior leads also to smaller correlation coefficients (*R* = 0.809 group α, *R* = 0.797 group β), if one performs a linear regression analysis in the nonlogarithmized data which implies a linear relationship: *c*_R_ = *a* + *bc*_I_. To conclude, this study supports that the relation between *c*_R_ and *c*_I_ should generally be described by equation (1) with *k* ≤ 1 and concomitantly *C*_0_ < 0.

This has consequences for further studies in the field of dust generation, since the linear relation *k* = 1 implies that a single process is responsible for a constant ratio of emission for both dust fractions over the entire range. On the other hand, the data of this study indicate that equation (1) or (2) are a better way to describe the dependencies of *c*_R_ and *c*_I_. One possible explanation for equation (1) or (2) are agglomeration effects which become more important with increasing concentrations (Barbosa-Cánovas *et al.*, 2005; Goudeli *et al.*, 2015). In addition, one can speculate that similar processes, which emit different concentrations of dust at different ratios, are attributed to the same working activity and material in the database. For example, the dust ratios generated by different brands of the same type of tool or by tools with different tear and wear are all attributed to the same working activity and material.

### Identification of groups

If one describes the data set by means of equation (1) or (2), one finds that the inhalable dust concentration is the single most important variable (adj. *R*^2^ = 0.585) for the respirable dust concentration: *k* = 0.594 and *C*_0_ = −0.990. The systematic inclusion of the variables *working activity* and *material* leads for example to the group 2-B (*high temperature processing* with *metal*), which is described by markedly different coefficients *k* = 0.759 and *C*_0_ = −0.687. All other groups in this systematic approach combine too many different dust generating processes and thus lead to coefficients similar to those of the total data set. Tables 2 and 3 demonstrate that it is important to go beyond such large groups, and that the subgroups α, β, γ, δ, and η, which are subgroups of group 2-B show a large variety of coefficients.

Unfortunately, there is no systematic way to form groups as in Table 3. One reason is that the technical information in the database includes only some aspects of the dust generating process. More specific information should be included such as the processing tools, grain sizes of sandpaper, types of grinding machines, or saw blades. The use of lubricants is another important example of missing information, since it reduces the friction and thus the amounts of particles generated by machining/abrasive techniques (Vaaraslahti *et al.*, 2005). The inclusion of this information could help to lead to a systematic identification of groups in the future.

### Application of results

Given the heterogeneity of formed groups, one has to be careful to use the model parameters in toxicological or epidemiological analyses without a careful check of applicability. For example, all results of this work are only valid for dust-generating processes in the German industry between 1998 and 2016 and the working conditions described in the preceding sections.

If one calculates ln(*c*_R_) from the regression coefficients in Table 2 for a given group and ln(*c*_I_), then the result has a confidence interval of ±1.96 · *s*_Fit_(ln(*c*_R_)). This variance has to be added to the other sources of uncertainty for the given data set of inhalable dust, such as measurement uncertainty and analytical uncertainty. In addition, one has to consider that the smaller value of *s*_Fit_ is only valid around the mean value of ln(*c*_I_).

The quality of the analysis is described by the correlation coefficient, which increases with increasing quality of the description. The best description of the data is given by groups α–η in Tables 2 and 3. For these groups the regression accounts for 73–83% of the variance in the data, and they constitute the main result of this study (adj. *R*^2^ from 0.733 to 0.835). Due to the detailed information on working activities and materials in Table 3 it may be possible to confirm the coefficients for groups α–η in experimental studies in the future.

For the estimation of the respirable fraction in other studies the authors recommend to use the conversion functions of the heuristic groups α–η in Tables 2 and 3. If the exposure condition in question cannot be found in this group one can resort to the combined groups 1-A to 6-B. If an assessment does not fit into these groups, the conversion functions of *working activity* (groups 1–6) or material (groups A–C) should be used, considering the larger uncertainty in these groups. As these groups are comprehensive it should always be possible to choose one of them and therefore it is not recommended to use the conversion function for the whole data set (group 0 in Table 2).

The main issue for the use of the conversion functions is to find a group that coincides with the exposure conditions in question. In going from the heuristic groups to the combined groups and the *working activity* or *material* group one is necessarily including exposure situations that differ from the one in question. Therefore, the proposed conversion functions are most useful in the context of average exposures for a large number of work places. Individual situations that are included in these large groups might differ significantly, and a careful consideration of the exposure conditions is more important than the analysis or the error terms in Table 2. It is well known, for example, that wood dust predominantly consists of inhalable dust. Therefore, it is not recommended to use the mineral-dominated material group A, although some wood measurements are included in its subgroup ‘others’. This would assume that wood dust is comparable to mineral-dominated dust, which is wrong. As a consequence, this study cannot make assumptions on the fraction of respirable wood dust.

### Comparison with literature

A comparison of the results of this analysis with other studies shows that the latter often assume a single factor for *c*_R_/*c*_I_ (i.e., a linear relation) and not a function such as equation (1). In any case, the present analysis can serve as additional information in studies like Dahmann *et al.* (2007), where data for inhalable and respirable dust in former uranium mines have been reconstructed by performing measurements with historic equipment.

Another example is the study of Jenkins *et al.* (2005), which shows that gas metal arc welding fume contains mainly particles <1 µm and thus a large prevalence of respirable dust. Other studies show an amount of respirable dust between 50 and 60% for various welding processes (Dasch and D’Arcy, 2008; Tsai *et al.*, 2011). The group γ, *welding*, confirms such amounts in the range of 0.65 mg m^−3^ ≤ *c*_I_ ≤ 1.55 mg m^−3^ using the coefficients of Table 2. In addition, we have found for group γ an adjusted *R*^2^ = 0.766 taking 9 different welding processes and 1126 parallel measurements into account. This corresponds to the results of Lehnert *et al.* (2012), who determined an adjusted *R*^2^ = 0.79 (for measurements using the GSP sampler) as a result of the multiple linear regression analysis considering five different welding processes and 241 measurements.

Notø *et al.* (2016) determined a ratio of *c*_R_/*c*_I_ ≈ 0.085 in ‘cement production’ with an adjusted *R*^2^ = 0.78 (*n* = 112). This includes *working activities* such as crushing, grinding, and milling. For these working conditions, we have only unspecific groups such as *machining/abrasive techniques* (4) or *mineral-dominated* (A) with coefficients: *k* ≈ 0.58, *C*_0_ = −1.0. For these coefficients a ratio of *c*_R_/*c*_I_ ≈ 0.085 is only possible for *c*_I_ > 30 mg m^−3^.

It is also not possible to determine heuristic groups like grinding gypsum and quartz sand, clay processing or loading cement as in earlier studies of the German Social Accident Insurance (Hauptverband der gewerblichen Berufsgenossenschaften, 1996). The number of measurements which are used during these early studies vary between 2 and 14, so the ratios of *c*_R_/*c*_I_ which have been determined are very specific for the respective measurement condition. The ratios 0.19 ≤ *c*_R_/*c*_I_ ≤ 0.26 of the earlier study are reached using the general coefficients of the whole data set: *k* ≈ 0.58, *C*_0_ = −1.0 in the range of 2.2 mg m^−3^ > *c*_I_ > 5.0 mg m^−3^.

## Summary and conclusion

In summary, it was possible to develop conversion functions for estimating the respirable out of the inhalable dust fraction on the basis of 15 120 data pairs. The amount of data which was analyzed, considering many different working activities and different types of material creates a good framework to support occupational hygienists and risk assessors and offer the opportunity to estimate respirable dust concentrations when only measurements of the inhalable fraction and enough information on the working scenario and the working material is available. With the given conversion functions it is possible to estimate missing concentrations for retrospective analyses which are often required for the assessment of occupational diseases or for epidemiological studies.

For the conversion functions, this study suggests that the data should generally be described by the equation (1) or (2) with *k* ≤ 1 and concomitantly *C*_0_ < 0. However, the equations yield a reasonable description only, if one chooses specific exposure conditions such as *working activities* and *material*.

With specific working conditions as described in Table 3, it is possible to identify groups α–η, where 73–83% of the variance in the data is accounted for by the regression functions described in Table 2. The results of the other groups in this study are less specific and therefore the estimation of respirable dust concentrations from inhalable dust measurements is associated with a larger uncertainty.

Fig. 4 and Table 2 show that each heuristic group has a different unique conversion function and the more information on the dust measurements is available for the calculation, the smaller is the error and the uncertainty.

For the evaluation of data in other studies the authors recommend to use the conversion functions of the heuristic groups α–η in Tables 2 and 3 and the combined groups 1-A to 6-B. When an assessment does not fit into these groups, the conversion functions of working activity (groups 1–6) or material (groups A–C) should be used, considering the larger uncertainty in these groups.

## Funding

The first author (C.W.) was financed by grant from the German Social Accident Insurance.

## References

[CIT0001] Andersson L, Bryngelsson IL, Ohlson CG et al (2009) Quartz and dust exposure in Swedish iron foundries. J Occup Environ Hyg; 6: 9–18. 18982534 10.1080/15459620802523943

[CIT0002] Ausschuss für Gefahrstoffe (2014) Begründung zum Allgemeinen Staubgrenzwert (2014/2001) in der TRGS 900 Vol. 4 pp. 1–25. Available at https://www.baua.de/DE/Angebote/Rechtstexte-und-Technische-Regeln/Regelwerk/TRGS/pdf/900/900-allgemeiner-staubgrenzwert.pdf?__blob=publicationFile. Accessed 10 December 2018.

[CIT0003] Barbosa-Cánovas GV, Ortega-Rivas E, Juliano P et al (2005) Food powders: physical properties, processing, and functionality. New York: Kluwer Academic/Plenum Publishers pp. 180–93. ISBN: 0-387-27613-0.

[CIT0004] Barig A, Blome H (1999) Allgemeiner Staubgrenzwert, Teil 1: Allgemeines. Gefahrst Reinhalt Luft; 59: 261–5.

[CIT0005] Baur X (2013) Berufskrankheiten der 4er-Gruppe der BKV-Anlage (Atemwege/Lunge). In: Budnik LT, Groth K, Oldenburg M, Popp W, and Wegner R, editors. Arbeitsmedizin. Heidelberg: Springer pp. 123–35.

[CIT0006] Bender F. H (2005). Sicherer Umgang mit Gefahrstoffen, Sachkunde für Naturwissenschaftler. Dritte, durchgehend aktualisierte Auflage Weinheim: Wiley-VCH pp. 55–9.

[CIT0007] Burstyn I, Teschke K, Kennedy SM (1997) Exposure levels and determinations of inhalable dust exposure in bakeries. Ann Work Expo Health; 41: 609–24. 10.1016/S0003-4878(97)00031-8 9375522

[CIT0008] Coenen W (1981) Beschreibung der Erfassungs- und Durchgangsfunktion von Partikeln bei der Atmung—messtechnische Realisierung. Staub Reinhalt Luft 41: 472–9.

[CIT0009] Dahmann D, Bauer HD, Stoyke G (2007) Retrospective exposure assessment for respirable and inhalable dust, crystalline silica and arsenic in the former German uranium mines of SAG/SDAG. Int Arch Occup Environ Health; 81: 949–58. 18071741 10.1007/s00420-007-0287-8

[CIT0010] Dasch J, D’Arcy J (2008) Physical and chemical characterization of airborne particles from welding operations in automotive plants. J Occup Environ Hyg; 5: 444–54. 18464098 10.1080/15459620802122720

[CIT0011] Deutsches Institut für Normung (2003) DIN 8580:2003. Fertigungsverfahren, Begriffe, Einteilung. Berlin: Deutsches Institut für Normung.

[CIT0012] Draper NR, Smith H (1998) Applied regression analysis. New York: Wiley & Sons. ISBN 0-471-17082-8.

[CIT0013] European Committee for Standardization (1993) EN 481:1993-09, Workplace atmospheres: size fraction definitions for measurement of airborne particles. Brussels: European Committee for Standardization.

[CIT0014] European Committee for Standardization (1995) EN 689:1995, Workplace atmospheres—guidance for the assessment of exposure by inhalation to chemical agents for comparison with limit values and measurement strategy. Brussels: European Committee for Standardization.

[CIT0015] European Committee for Standardization (2012) EN 1540:2012-03, Workplace exposure—terminology. Brussels: European Committee for Standardization.

[CIT0016] European Committee for Standardization (2014a) EN 13205 Part 1–6, Workplace exposure—assessment of sampler performance for measurement of airborne particle concentrations. Brussels: European Committee for Standardization.

[CIT0017] European Committee for Standardization (2014b) EN: 13205-2:2014, Workplace exposure—assessment of sampler performance for measurement of airborne particle concentrations—Part 2: Laboratory performance test based on determination of sampling efficiency. Brussels: European Committee for Standardization.

[CIT0018] Gabriel S, Koppisch D, Range D (2010) The MGU—a monitoring system for the collection and documentation of valid workplace exposure data. Gefahrst Reinhalt Luft; 70: 43–9.

[CIT0019] Goudeli E, Eggersdorfer ML, Pratsinis SE (2015) Coagulation-agglomeration of fractal-like particles: structure and self-preserving size distribution. Langmuir; 31: 1320–7. 25560979 10.1021/la504296z

[CIT0020] Hahn J-U, Möhlmann C (2011) Neuer A-Staub-Grenzwert—Aspekte für dessen Anwendung. Gefahrst Reinhalt Luft; 71: 429–32.

[CIT0021] Hauptverband der gewerblichen Berufsgenossenschaften (1996) Stäube an Arbeitsplätzen in der DDR—Umrechnungsfaktoren der Meßverfahren. In Ziem H, Plitzko S, Thürmer H, Pfeiffer W, Kupfer J, editors. Meßergebnisse für mineralische (asbestfreie) Stäube, Bewertung; BIA-Report 5/96. BIA-Report, Sankt Augustin: Hauptverband der gewerblichen Berufsgenossenschaften; pp. 62–78.

[CIT0022] International Organization for Standardization (1995) ISO 7708:1995, Air quality—particle size fraction definitions for health-related sampling. Geneva: International Organization for Standardization.

[CIT0023] Janssen J, Laatz W (2017). Statistische Datenanalyse mit SPSS: Eine anwendungsorientierte Einführung in das Basissystem und das Modul Exakte Test. Berlin: Springer Gabler Verlag. ISBN 978-3-662-53476-2.

[CIT0024] Jenkins NT, Pierce WM-G, Eagar TW (2005) Particle size distribution of gas metal and flux cored arc welding fumes. Weld J; 84: 156–63.

[CIT0025] Korczynski RE (2011) Dust exposures and ventilation control in the Crematorium. Appl Occup Environ Hyg; 12: 122–5.

[CIT0026] Lehnert M, Pesch B, Lotz A et al; Weldox Study Group. (2012) Exposure to inhalable, respirable, and ultrafine particles in welding fume. Ann Occup Hyg; 56: 557–67. 22539559 10.1093/annhyg/mes025 PMC3387834

[CIT0027] Lilienberg L, Brisman J (1994) Flour dust in bakeries—a comparison between methods. Ann Occup Hyg; 38: 571–5. 10.1093/annhyg/38.1.67 8161093

[CIT0028] Linnainmaa M, Laitinen J, Leskinen A et al (2007) Laboratory and field testing of sampling methods for inhalable and respirable dust. J Occup Environ Hyg; 5: 28–35. 10.1080/15459620701763723 18041642

[CIT0029] Martin JR, Zalk DM (2011) Comparison of total dust/inhalable dust sampling methods for the evaluation of airborne wood dust. Appl Occup Environ Hyg; 13:177–82.

[CIT0030] Mattenklott M., Möhlmann C (2011) Probenahme und analytische Bestimmung von granulären biobeständigen Stäuben (GBS). Gefahrs Reinhalt Luft; 10: 425–8.

[CIT0031] Möhlmann C (2005) Staubmesstechnik—damals bis heute. Gefahrst Reinhaltr Luft; 5: 191–4.

[CIT0032] Notø HP, Nordby KC, Eduard W (2016) Relationships between personal measurements of ‘total’ dust, respirable, thoracic, and inhalable aerosol fractions in the cement production industry. Ann Occup Hyg; 60: 453–66. 26755796 10.1093/annhyg/mev093

[CIT0033] Riediger G (2001) Geräte zur Probenahme der einatembaren Staubfraktion (E-Staub). Messung von Gefahrstoffen, IFA-Arbeitsmappe; Erich Schmidt Verlag: 27/2001, 3010 Available at https://www.ifa-arbeitsmappedigital.de/IFA-AM_3010. Accessed 07 May 2019.

[CIT0034] Sachs L (1999). Angewandte Statistik. Berlin-Heidelberg-New York: Springer Verlag. ISBN 978-3-662-05750-6.

[CIT0035] Siekmann H (1998) Geräte zur Probenahme der alveolengängigen Staubfraktion (A-Staub). Messung von Gefahrstoffen, IFA-Arbeitsmappe; Erich Schmidt Verlag: 21/1998, 3020 Available at https://www.ifa-arbeitsmappedigital.de/IFA-AM_3020. Accessed 07 May 2019.

[CIT0036] Tsai PJ, Vincent JH, Wahl G et al (1995) Occupational exposure to inhalable and total aerosol in the primary nickel production industry. Occup Environ Med; 52: 793–9. 8563841 10.1136/oem.52.12.793 PMC1128379

[CIT0037] Tsai P-J, Werner MA, Vincent JH et al (2011) Worker exposure to nickel-containing aerosol in two electroplating shops: comparison between inhalable and total aerosol. Appl Occup Environ Hyg; 11: 484–92.

[CIT0038] Vaaraslahti K, Keskinen J, Giechaskiel B et al (2005) Effect of lubricant on the formation of heavy-duty diesel exhaust nanoparticles. Environ Sci Technol; 39: 8497–504. 16294893 10.1021/es0505503

[CIT0039] Verma DK (1984) Inhalable, total and respirable dust: a field study. Ann Occup Hyg; 28: 163–72. 6476684 10.1093/annhyg/28.2.163

[CIT0040] Vinzents PS, Thomassen Y, Hetland S (1995) A method for establishing tentative occupational exposure limits for inhalable dust. Ann Occup Hyg; 39: 795–800. 8588714 10.1016/0003-4878(95)00036-4

[CIT0041] Weggeberg H, Føreland S, Buhagen M et al (2016) Multi-element analysis of airborne particulate matter from different work tasks during subsea tunnel rehabilitation work. J Occup Environ Hyg; 13: 725–40. 27078031 10.1080/15459624.2016.1177645

[CIT0042] Werner MA, Spear TM, Vincent JH (1996) Investigation into the impact of introducing workplace aerosol standards based on the inhalable fraction. Analyst; 121: 1207–14. 8831279 10.1039/an9962101207

[CIT0043] WHO—Occupational and Environmental Health Department of Protection of the Human Environment World Health Organization (1999) WHO/SDE/OEH/99.14, Hazard prevention and control in the work environment: airborne dust. Geneva: Occupational and Environmental Health Department of Protection of the Human Environment World Health Organization.

